# Stochastic Variation in DNA Methylation Modulates Nucleosome Occupancy and Alternative Splicing in *Arabidopsis thaliana*

**DOI:** 10.3390/plants11091105

**Published:** 2022-04-19

**Authors:** Ibtissam Jabre, Saurabh Chaudhary, Cornelia M. Wilson, Dorothee Staiger, Naeem Syed

**Affiliations:** 1 School of Biosciences and Medicine, University of Surrey, Guildford GU2 7XH, UK; 2 School of Biosciences, Cardiff University, Cardiff CF10 3AX, UK; chaudharys6@cardiff.ac.uk; 3 Life Sciences Industry Liaison Lab, School of Psychology and Life Sciences, Canterbury Christ Church University, Sandwich CT13 9ND, UK; cornelia.wilson@canterbury.ac.uk; 4 RNA Biology and Molecular Physiology, Faculty of Biology, Bielefeld University, 33615 Bielefeld, Germany; dorothee.staiger@uni-bielefeld.de; 5 School of Human and Life Sciences, Canterbury Christ Church University, Canterbury CT1 1QU, UK

**Keywords:** *Arabidopsis thaliana*, stochastic DNA methylation, alternative splicing, cold stress, co-transcriptional alternative splicing

## Abstract

Plants use complex gene regulatory mechanisms to overcome diverse environmental challenges. For instance, cold stress induces rapid and massive transcriptome changes via alternative splicing (AS) to confer cold tolerance in plants. In mammals, mounting evidence suggests chromatin structure can regulate co-transcriptional AS. Recent evidence also supports co-transcriptional regulation of AS in plants, but how dynamic changes in DNA methylation and the chromatin structure influence the AS process upon cold stress remains poorly understood. In this study, we used the DNA methylation inhibitor 5-Aza-2′-Deoxycytidine (5-aza-dC) to investigate the role of stochastic variations in DNA methylation and nucleosome occupancy in modulating cold-induced AS, in *Arabidopsis thaliana* (Arabidopsis). Our results demonstrate that 5-aza-dC derived stochastic hypomethylation modulates nucleosome occupancy and AS profiles of genes implicated in RNA metabolism, plant hormone signal transduction, and of cold-related genes in response to cold stress. We also demonstrate that cold-induced remodelling of DNA methylation regulates genes involved in amino acid metabolism. Collectively, we demonstrate that sudden changes in DNA methylation via drug treatment can influence nucleosome occupancy levels and modulate AS in a temperature-dependent manner to regulate plant metabolism and physiological stress adaptation.

## 1. Introduction

Plants employ different genetic and epigenetic strategies to fine-tune their transcriptional response during daily cycles and under stress to support life and confer adaptive responses [1,2]. Emerging evidence demonstrates that co-transcriptional alternative splicing (AS) is a key gene regulatory mechanism in plants [3,4,5]. Unlike constitutive splicing, AS of pre-mRNA uses alternative splice sites to generate multiple transcripts from a single gene, and it manifests in four major types, including exon skipping (ES), intron retention (IR), the alternative 3′ splice site (A3′ SS), and the alternative 5′ splice site (A5′ SS), of which IR is the most prevalent event in plants [4].

In plants, up to 70% of intron-containing genes are alternatively spliced, contributing towards transcriptome diversity and potentially proteome complexity [6,7,8]. AS also plays an important role in response to abiotic and biotic stresses during different development stages [9,10,11,12,13] and in regulating the transcript isoform levels of key circadian clock genes in *Arabidopsis thaliana* (Arabidopsis) [14,15].

Regulation of AS is orchestrated by the abundance of multiple transacting factors including splicing factors (SFs) recognizing various *cis*-regulatory elements within the RNA sequences in a cell type- and condition-dependent manner [16,17]. Nevertheless, DNA sequences located upstream or within the coding region can also impact splicing outcomes. From this perspective, emerging evidence has shown that the chromatin environment, such as DNA methylation and nucleosome occupancy, also affect RNA polymerase II (RNAPII) processivity and co-transcriptional splicing and SFs recruitment [18,19,20,21,22]. In eukaryotes, DNA methylation occurs in symmetric CG and CHG (H = A, T or C) and asymmetric CHH contexts [23]. However, DNA methylation is largely dependent on the CpG context in plants. In the Arabidopsis genome, 24% of CG sites are methylated, compared with only 6.7% of CHG and 1.7% of CHH sites [24,25]. Constitutive exons have higher CG methylation content and nucleosome occupancy levels compared to introns and alternative exons in animals and plants [26,27,28,29,30,31,32,33]. DNA methylation is also higher in nucleosome-bound DNA in both humans and Arabidopsis affecting chromatin compaction/remodelling [26,29,34]. It is not surprising that AS is largely a co-transcriptional process, as RNA polymerase II (RNAPII) speed is affected by the chromatin state, which in turn affects splicing outcomes [35,36,37]. Native elongating transcript sequencing (NET-seq) and global run-on sequencing (GRO-seq) data from Arabidopsis show that phosphorylation of the RNAPII C-terminal domain (CTD) mediates interactions with the spliceosome and that RNAPII accumulation is associated with different chromatin states [37].

Plants also exhibit stable as well as dynamic DNA methylation patterns under different growth and stress conditions that provide the template to modulate gene expression and AS in a condition-specific manner [38,39,40,41,42]. Deregulation of DNA methylation has been involved in multiple diseases such as cancer and genomic instability [43,44]. However, in plants, no studies have shed light on the sudden changes in DNA methylation levels; in particular, DNA hypomethylation can affect plants’ stress responses AS. Here, we used 5-Aza-2′-Deoxycytidine (5-azad-C) to induce DNA hypomethylation in the Arabidopsis Columbia (Col-0) ecotype. 5-aza-dC is a nucleoside analogue of cytosine that inhibits DNA methyltransferases, resulting in hypomethylation and gene activation through the uncoiling of constitutive heterochromatin [45]. 5-aza-dC has also been demonstrated to create a heritable hypomethylation and phenotypic trait variation in rice [46,47], flax [48], tobacco [49], *Brassica* [50], *Melandrium album* [51], triticale [52], Arabidopsis [53], *Fragaria vesca* [54], and *Solanum ruiz-lealii* [55].

In Arabidopsis, a cold temperature induces a cascade of gene expression reprogramming to modulate the gene, transcript, and proteome level to regulate multiple aspects of physiological processes for stress adaptation [56,57,58]. Collective data demonstrate that AS is the hub of cold-stress responses in plants as part of gene expression regulation at the transcript level [9,12,59]. For example, in Arabidopsis, cold-dependent AS of the *LATE ELONGATED HYPOCOTYL (LHY)* gene generates different transcripts with variable abundance [14], and recently, co-transcriptional regulation of *LHY* pre-mRNA splicing under cold stress has been proposed to be regulated by the chromatin structure [5]. Moreover, cold-induced DNA methylation and nucleosome occupancy changes are relatively rapid epigenetic regulators that mediate environmental cues and provide flexible cold responses in rice, Arabidopsis, and maize [42,60,61,62,63]. We recently demonstrated [22] that stable epigenetic variations with a similar genetic background are sufficient to induce changes at the AS level in Arabidopsis. Hence, here we aimed to understand how 5-aza-dC and cold stress induced a variation in DNA methylation, how nucleosome occupancy modulates gene expression and AS, and the physiological impact of such regulation.

## 2. Materials and Methods

### 2.1. Plant Growth and Chemical Treatments

Arabidopsis Col-0 ecotype seeds were surface-sterilised with 30% (*v*/*v*) household bleach and 5% (*v*/*v*) Triton X-100 for 10 min and then washed seven times with distilled water. Sterilised seeds were air-dried and sown on Petri plates containing agar medium. For control plants, the medium was comprised of the Murashige and Skoog basal salt mixture, 2-(N-Morpholino) ethanesulfonic acid (MES), sucrose, and Gamborg’s Vitamin Solution. For 5-aza-dC treatment, 5-aza-dC (dissolved in 50% (*v*/*v*) acetic acid) was added to a final concentration of 4 ug/mL to agar medium. Plates were wrapped with parafilm, incubated for 4 days at 4 °C to synchronise germination, and then transferred to a growth chamber and grown using 16-h light/8-h dark, 22 °C, and 50% relative humidity. One week after, plants were transferred to soil pots under the same conditions. After three weeks, leaf tissues (3 replicates for each condition) were harvested from plants grown at 22 °C and plants subjected to 24 h of cold treatment (4 °C). Tissue was flash-frozen in liquid N_2_ and stored at −80 °C until the isolation of RNA, genomic DNA (gDNA), and nucleosome gDNA, as described below.

### 2.2. RNA and DNA Extraction

Total RNA and gDNA was extracted from Arabidopsis leaf tissue using the RNeasy and DNeasy Plant Mini Kits (Qiagen, cat n^o^ 69204), respectively. Purified RNA and DNA was treated with DNase and RNase for library preparation, respectively, as described below.

### 2.3. Nuclei Isolation and Digestion with MNase

Briefly, 2 g of leaf tissues were ground into powder in liquid N_2_. Afterwards, the nuclei are purified with a nuclei extraction buffer (0.25 M sucrose, 60 mM KCl, 15 mM MgCl_2_, 1 mM CaCl_2_, 15 mM PIPES (pH 6.8), 0.8% (*v*/*v*) Triton X-100, 1 mM PMSF), digested with 0.5 units/µL micrococcal nuclease (MNase) (NEB) for 10 min. Finally, purified nuclei are incubated with Proteinase K overnight at 37 °C.

### 2.4. Library Preparation and Sequencing Information

For RNA-seq, the libraries were constructed starting with 1 μg of total RNA using the TruSeq RNA protocol (Illumina cat n^o^ 15026495 Rev.F) Libraries were sequenced on the Hiseq 4000 (Illumina) using 150 paired end reads across 3 lanes. The total number of raw reads generated in the RNA-seq data were ~23 M per biological replicate for each sample. For WGBS, libraries were prepared starting with 1 μg of genomic DNA using a KAPA high throughout Library Prep Kit with amendments (Part No: KK8234). Libraries were sequenced on the HiSeq 4000 using v1 chemistry and 150 bp paired end reads over 2 lanes. The total number of raw reads generated in the WGBS-seq data were ~23 M per biological replicate.

For the MNase-seq libraries, mononucleosome-sized DNA fragments were selected using Beckman Coulter XP beads (Beckman Coulter cat n^o^ A63880), followed by library preparation using NEBNext^®^ Ultra™ II DNA library kit Prep for Illumina^®^ (New England BioLabs, Ipswich, MA, USA, NEB #E7103). Briefly, 1 μg of fragmented DNA fragments were ligated to paired-end adaptors after blunt-ended treatment. Size selection of Adaptor ligated DNA was then performed using AMPure XP beads. A final PCR enrichment step of the adaptor-ligated DNA was performed followed by a clean-up of the PCR reaction. The insert size of the libraries (~150 bp) was verified by running an aliquot of the library on the Agilent bioanalyser using the High Sensitivity chip (Agilent cat n^o^ 5067–4626) and the concentration was determined by using a High Sensitivity Qubit assay (ThermoFisher cat n^o^ Q32854).

### 2.5. DE and DAS Analysis

Low quality RNA-Seq reads with Phred quality score values lower than 30 are filtered from the raw data using Trimmomatic [64]. Salmon version 0.82 was run using AtRTD2-QUASI to quantify the expression of transcripts from RNA sequencing [65,66]. AtRTD2-QUASI is an accurate reference transcript dataset for Arabidopsis Ctrl and has been used previously for the accurate quantification of individual transcript abundance for alternative splicing analysis. First, the Arabidopsis reference dataset was indexed using the quasi-mapping mode (type quasi) of Salmon to create k-mers of lengths 31. For quantification, the input data sequence bias correction and bootstrapped abundance estimate are performed using the “seqBias” and “nom-Bootstraps 30” parameters from Salmon, respectively. Differential expression (DE) and differential alternative splicing (DAS) are carried out as described previously by [12]. Briley, the “lengthScaledTPM” method in the tximport version 1.10.1 was used to generate transcript and gene level read counts and TPMs from Salmon outputs [67]. For each biological replicate, technical replicates run in the different sequencing lane are summed up to increase sequencing depth. Then, low expressed transcripts are filtered out based on the criteria that an expressed transcript must have an expression of ≥1 counts per million (CPM) in at least 2 out of 24 samples. Hence, expressed genes are the genes with at least one transcript passing the expression level filtering criteria. The library size of each sample was then normalised using trimmed mean of M values (TMM) in edgeR (version 3.12.1) [68,69]. The principal component analysis demonstrated no batch effect within the three biological replicates. Normalised read counts in gene and transcript levels are transformed into log_2_CPM using the Limma package version 3.26.9 [70]. To detect differential expression at the gene and transcript levels, six contrast groups are set up using temperature and treatment. A gene was considered significantly DE if the log_2_ fold changes of CPM for each contrast group was ≥1 and *p* values (adjusted by the Benjamini-Hochberg method for multiple testing correction) < 0.01. To detect DAS genes, the log_2_ fold changes of each transcript was compared to the weighted average of log2 fold changes of all transcripts of the gene, which is a proxy of gene level changes. A F-test is carried out to test whether the changes for all the transcripts and the gene are the same. A gene was classified as significantly DAS if *p* value < 0.01 and if the difference in the relative abundance of an alternative splice isoform in relation to the total gene expression within a contrast group (ΔPS) ≥ 0.1.

### 2.6. Parametric Gene Set Enrichment Analysis

To perform the PGSEA enrichment (PAGE), the PGSEA package has been used to display the activity of pathways in individual samples in terms of Z scores, characterizing the mean of the fold-changes for all genes in a certain pathway. Analysis of variance (ANOVA) on the Z scores across sample groups has been applied and after selecting a cut-off with FDR < 0.05, pathways are ranked by the standard deviation, and only the top 30 pathways are represented in the heatmap.

### 2.7. Gene Ontology Analysis

Gene functional enrichment analysis was performed on differentially expressed and alternatively spliced genes using the Database for Annotation, Visualization, and Integrated Discovery (DAVID version 6.8) with default parameters [71,72]. The gene ontology (GO) terms (biological process, molecular function, and cellular components) were identified to provide biological insights into the significance of DE and DAS using an FDR ≤ 0.05.

### 2.8. Identification of AS Events, PSI, and Delta PSI from RNA-Seq Data

To identify AS events, we quantified transcript abundance in TPM using Salmon [65]. Then, SUPPA2 [73] was used to identify the different AS events from the AtRTDv2 GTF annotation file [74]. Then, the percentage spliced in PSI for each event was calculated as the ratio of the TPM of transcripts that contribute to the event over the TPM of transcripts of the same gene. Differential splicing of local events between multiple conditions was calculated as the difference of the mean PSI between conditions for each event, and the *p*-value of this difference.

### 2.9. MNase-Seq Data Processing

Paired-end MNase-seq reads are mapped to the TAIR.10 Arabidopsis reference genome using Bowtie version 1.2.2 [75], in which the setting “-m” was set to 1 to output only uniquely mapped reads. A 3-column BED file for iNPS for accurate genome-wide nucleosome positioning (chromosome, starting coordinate, and ending coordinate for each tag) [76]. A wave-form nucleosome signal profile was generated from tag coordinate bed data (nucleosome scoring), which are further smoothened using discrete Gaussian convolution. Three Gaussian derivatives are then performed to detect important sites on the smoothed wave-form profile (max/min-extremum points, inflection points, and most winding positions). Nucleosome were then classified as main, or shoulder-based on the filtering criteria described previously by [76]. Low quality nucleosomes are filtered out based on six criteria implemented by the iNPS algorithm [76]. The confidence level of detected nucleosome was calculated using both the upper- and lower-tailed Poisson tests, in which the first test identifies tag enrichment within the peak region and the second identifies the tag depletion within the adjacent ‘valley’ regions flanking the corresponding nucleosome. This results in two respective scores ‘−log10 (*p*-value_of_peak)’ and ‘–log10 (*p*-value_of_valley)’ for each detected nucleosome. For each set of genes, the average ‘nucleosome raw profiles’ for exons are generated by combining the profiles 2 kb upstream of the transcription start site (TSS), gene body, and transcription terminal site (TTS). For the analysis of gene expression level against the nucleosome in genes, the expression level was based on TPM values for each gene derived from Salmon. The genes are divided into five groups based on their TPM values.

### 2.10. Differential Nucleosome Positioning

For differential nucleosome positioning, for only tags having their mid-point locating with any nucleosome peak, the corresponding tags (chromosome, start, and end) are selected and inputted into DANPOS version 2 [77]. DANPOS was run with the parameters as described previously by INPs. ‘-q,--height’ = 1 (the intensity cut off for nucleosome calling), ‘-z,--smooth_width’ = 100 (the smooth width before peak calling), ‘-e,--edge’ = 1 (detect edges for peaks), ‘-k,--keep’ = 1 (saving mid-stage files), ‘-x,--pcfer’ = 0 (no nucleosome calling), ‘-n,--nor’ = N (no normalization), ‘--frsz’ = 150 (setting the average size of DNA fragment to 150 bp), and ‘--clonalcut’ = 0 (do not adjust clonal signal). DANPOS scores the difference of the nucleosome signal between two samples of each contrast group using *p*-values and false positive rates (FDRs); hence, significantly different nucleosomes are selected only if ‘point_diff_FDR’ ≤ 0.01 and ‘smt_diff_FDR’ ≤ 0.05. Then, a 2000-bp sliding window was moved across the genome with a 500 bp step size to select the windows enriched with differentially positioned nucleosomes (~ top 1% windows that have ≥2 differentially positioned nucleosomes are selected). Plant Biomart was then used to identify genes associated with DPNs in selected windows.

### 2.11. Bisulphite-Sequencing Data Processing

The sequence data were filtered for adapter sequences and low quality reads using Trimmomatic V0.32 [64] with default parameters. The quality filtered reads from each sample are aligned to the Arabidopsis TAIR.10 reference genome using Bismark [78], allowing only one mismatch per seed. Uniquely aligned reads are retained to remove potential clonal bias due to PCR amplification and also remove duplicate reads using Bismark v0.15.0. 1 [78]. The mapped reads on the genome are used as inputs in the methylKit version v1.10.0 [79], in order to estimate the efficiency of bisulphite conversion that shows a bisulphite conversion efficiency of ≥96% achieved for all the samples. All splice junctions are stacked (100 bp exon + 100 bp intron for the donor, 100 bp intron + 100 bp exon for the acceptor), then the methylation level of each base pair was calculated as C/(C+T) from the first nucleotide of both strands. Methylation profiles for exons are generated by combining the profiles 2 kb upstream of the transcription start site (TSS), gene body, and transcription terminal site (TTS). For the analysis of gene expression level against methylation in genes, the expression level was based on the TPM values for each gene derived from Salmon. Genes are divided into five groups based on their TPM values.

## 3. Results

### 3.1. 5-Aza-2′-Deoxycytidine Treatment Affects Gene Expression and Alternative Splicing Profiles

**Figure 1:**
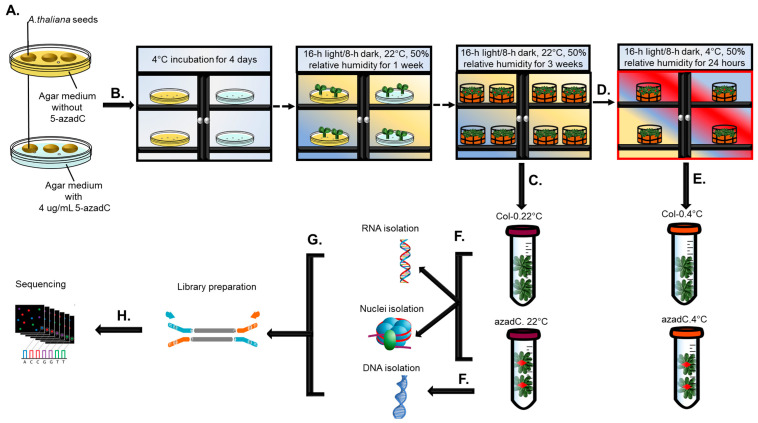
Summary of the experimental procedure. (**A**) *Arabidopsis thaliana* (*A. thaliana*) sown on agar plates with/without 5-azadC. (**B**) Germination synchronisation, followed by incubation at *A. thaliana* growth conditions. (**C**) Leaf sample collection from Col-0 and azadC plants grown at 22 °C. (**D**) Cold stress treatment for 24 h. (**E**) Leaf sample collection from Col-0 and azadC plants grown at 4 °C. (**F**) RNA and nuclei isolation from azadC and Col-0 plants grown at 22 °C and plants shifted to cold stress, and DNA isolation from azadC plants grown at 22 °C and plants shifted to cold stress. (**G**) Library preparation followed by (**H**) Illumina HiSeq 4000 sequencing. Red asterisk indicates plants treated with 5-aza-dC.

To investigate the effect of 5-aza-dC treatment on gene expression and AS profiles under normal (22 °C) and cold (4 °C) conditions, we generated RNA sequencing (RNA-seq) data from Arabidopsis (Col-0 ecotype) with or without 5-aza-dC treatment on agar plates (azadC) with three biological replicates before and after their shift from 22 °C to 4 °C for 24 h (Figure 1).

We applied the stringent filtering criterion of 3D RNA-seq pipeline to identify differentially expressed (DE) and alternatively spliced (DAS) genes at the gene and transcript levels, respectively [63] (Table S1). Principal component analysis (PCA) across all samples demonstrated that both temperature (71.3% and 59% of total variance, respectively) and 5-aza-dC treatment (10.6% and 9% of total variance, respectively) are the contributors to gene expression variation at the gene and transcript levels (Figure S1a).

**Figure 2:**
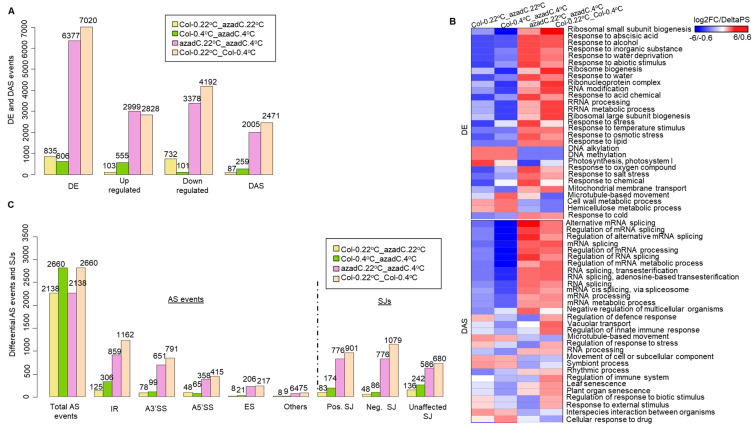
RNA-Seq data analysis summary in different contrast groups. (**A**) Histogram representing the number of differentially expressed (DE) genes categorised into up-regulated (**Up_regulated**) and down-regulated (**Down_regulated**), and differentially alternatively spliced (DAS) genes detected by the 3D-RNA seq pipeline. The x-axis represents DE and DAS genes count detected in each contrast group, and the y-axis represents the count number detected in each analysis. (**B**) Parametric gene set enrichment analysis defining biological processes’ gene ontology (GO) terms of enrichment in each contrast group for differentially expressed (DE) and differentially alternatively spliced (DAS) genes. Red and blue indicates activated and suppressed GO terms, respectively. (**C**) Histogram representing the number of different AS events and the directionality of splice junctions (SJs) in each contrast group detected by SUPPA. The x-axis represents AS events and SJs count detected in each contrast group, and the y-axis represents the count number detected in each analysis.

RNA-seq data analysis at the gene level show that treatment with 5-aza-dC resulted in less DE genes under cold stress compared to normal growth conditions (835 and 606 DE genes at 22 °C and 4 °C, respectively: Figure 2A). Conversely, at the transcript level, 5-aza-dC treatment results in a dramatic increase in the splicing of genes under cold stress compared to normal growth conditions (87 and 259 DAS genes at 22 °C and 4 °C, respectively: Figure 2A). These data indicate that changes in DNA-methylation caused by 5-aza-dC treatment are more likely to affect gene expression at the gene and transcript levels independently upon cold stress. Further, we have detected more changes at the gene and transcript level in Col-0 (7020 and 2471 genes, respectively) plants compared to azadC (6377 and 2005 genes, respectively) upon their shift from 22 °C to 4 °C. This indicates that 5-aza-dC treatment results in hypomethylation [52], and loose chromatin structure, which may result in a lower number of DAS genes and higher transcription efficiency. Indeed, we detected the highest number of up-regulated genes for azadC plants upon their shift to cold stress (2999 up-regulated genes) (Figure 2A). This is in line with previous reports from Arabidopsis demonstrating that DNA demethylation by 5-azadC can lead to the reactivation of silenced genes [80]. Nevertheless, this was not the case for Col-0 plants upon their shift from 22 °C to 4 °C, where the number of down-regulated genes is almost twice compared to azadC plants (4192 and 2828 genes; respectively). Moreover, we could detect that the number of DAS genes was lower for azadC plants compared to Col-0 upon their shift to cold stress (2471 and 2005 genes; Table S1). DE and DAS genes detected in each contrast group demonstrated an overlap percentage between 0.8% to 10% only (Figure S1b). Moreover, the overlap for DAS and DE genes between all contrast groups was only 1% (Figure S1c), implying that the DNA-dependant regulation of gene expression is temperature dependant.

Parametric Gene Set Enrichment algorithm (PGSEA) within the PGSEA package was used for DE and DAS gene detection in each group (Table S2). PGSEA of DE genes demonstrate that azadC treatment down-regulates the expression of genes related to key biological functions and up-regulates the expression of stress-related genes, whereas the PGSEA of DAS genes demonstrates a decrease in the splicing variation of splicing-related genes upon azadC treatment, which are the most suppressed pathways (Figure 2B). Nevertheless, we detected that cold stress induces the activation of various stress- and splicing-related pathways, which varied in expression between azadC and Col-0 plants (Figure 2B). Similarly, the PGSEA of DE and DAS genes demonstrate distinct enrichment of the GO term in the category of molecular functions and cellular components for azadC plants and Col-0 (Figure S2). For DE genes, we could detect that the DNA-binding transcription factor activity and DNA-binding pathways are enriched at the molecular level for genes that are differentially regulated upon azadC treatment under cold stress (Figure 2A). Similarly, for DAS genes, pathway analysis at the molecular level shows an enrichment in spliceosome complexes for azadC plants after cold treatment, whereas the ribonucleoside and GTP-binding activity seems to be abolished for azadC plants compared to Col-0 plants.

Overall, our data show the importance of DNA methylation in regulating key physiological processes in response to cold stress, and that genome-wide hypomethylation in plants may result in variable splicing patterns. These data indicate that DNA methylation is essential for canonical splicing regulation and may regulate gene expression at the gene and transcript levels independently, which is in line with a previous report from Arabidopsis [12].

### 3.2. Stochastic DNA Methylation Affects Splice Junctions and AS Event Profiles

As our DAS analysis demonstrates that 5-aza-dC treatment results in lower DAS changes alongside the up-regulation of gene expression in a temperature dependant manner, we hypothesised that DNA methylation may be important in defining intron-exon boundaries, RNAPII processivity, and spliceosome recruitment. Towards this goal, local AS events Percentage Spliced In (PSI) values for a total of 43,953 AS events and the difference of their distribution (ΔPSI) have been identified by SUPPA, which also was used as a reference to define the positively, negatively, and unaffected SJs in azadC and Col-0 plants under 22 °C and 4 °C (Table S3).

Interestingly, like our DAS analysis, the overall number of differentially regulated AS events upon 5-aza-dC treatment decreases upon cold stress (2138 AS events) compared to normal growth condition (2660 AS events, Figure 2C, AS events). This shows that DNA methylation is essential for AS events regulation and that DNA methylation differentially regulates AS events upon cold stress treatment compared to normal growth conditions. These findings are further confirmed by our classification of SJs in the different contrast groups, where azadC treated plants show an overall lower number of affected SJs in all three contexts (776 positively affected, 776 negatively affected, and 586 unaffected SJs) compared to Col-0 (901 positively affected, 1079 negatively affected, and 680 unaffected SJs) upon their shift to 4 °C (Figure 2C, SJs, Table S3). As expected, IR events are the most prevalent AS event influenced by methylation changes and/or cold stress followed by A5′SS and A3′SS, whereas ES was the least observed. This is similar to the overall frequency of AS events previously observed in Arabidopsis [6]. Thus, the changes in DNA methylation affect splicing events, yet does not affect the frequency of local AS events under normal growth and cold stress conditions. This can be potentially through the different organisation of chromatin structure around the splice junction, which can subsequently affect the recognition of splice sites by the splicing machinery.

### 3.3. DNA Methylation Fine-Tunes Gene Expression

**Figure 3:**
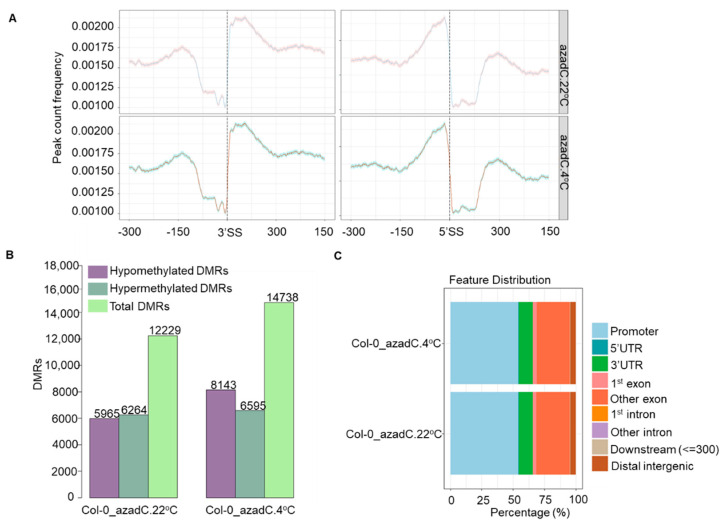
Bisulphite-Seq data analysis summary. (**A**) DNA methylation profiles across splice sites for azadC plants at 22 °C (**top panel**) and 4 °C (**lower panel**) in the CpG context. The x-axis represents 5′SS and 3′SS alongside 300 bp upstream (−0.3 kb) and downstream (0.3 kb) from the splice sites. The y-axis represents the DNA methylation count frequency with a confidence interval (0.95) estimated by bootstrap method. (**B**) Histogram representing hc_DMRs’ count number for the different methylation contexts detected between Col-0 and azadC at 22 °C (**left**) and 4 °C (**right**). (**C**) Percentage of CpG DMR distribution over different genomic features for different contrast groups.

To investigate how stochastic DNA methylation affects the gene expression and AS regulation observed in our RNA-seq analysis, we performed whole-genome bisulphite sequencing (WGBS) for azadC plants grown at 22 °C and 4 °C. Methylkit [79] was used to obtain genome-wide DNA methylation base calls. In line with previous studies from plants [22,26,81] and animals [30], we also observe higher methylation levels around exons compared to introns for the methylated CpG dinucleotides (mCpG) as well as CHG and CHH contexts (Figure 3A and Figure S3a,b). We also detected a sharp drop in methylation level for all methylation contexts at both splice sites (3′SS and 5′SS) (Figure 3A and Figure S3a,b).

As our PGSEA demonstrates that stress- and splicing-related pathways are down-regulated upon azadC treatment (Figure 2B), we aimed to illuminate what the differentially methylated regions between azadC and Col-0 are that could be involved in such a regulation. Towards this goal, we identified highly confident differentially methylated regions (hc_DMRs; Fisher’s exact test, *p*-value ≤ 0.01) for all three contexts of CpG, CHG, and CHH, in azadC at 22 °C and 4 °C in comparison to 54 Columbia (Col) lines of Arabidopsis using the hcDMR caller pipeline developed by the Jacobsen group at the University of California [82] and as described previously by [22]. We identified a total of 12229 and 14738 hc_DMRs upon azadC treatment under normal and cold stress; respectively (Figure 3B, Tables S5–S10). We observed that the number of hypomethylated regions as well as the total number of DMRs increase under cold stress compared to normal growth conditions, which is in line with previous reports showing that global levels of DNA methylation drop in plants under stress conditions [60]. The increase in hypomethylated DMRs upon cold stress also supports our previous observations (Figure 2B) related to pronounced decreases in splicing- and stress response- related pathways.

Further, we performed a genomic feature distribution of the DMRs to reveal how stochastic DNA methylation changes could affect splicing-related pathways. Interestingly, we found that CG DMRs are largely distributed over promoters and exons (Figure 3C), and hence reflect the decrease in splicing detected for azadC plants compared Col-0 at both temperature treatments (Figure 2B). Nevertheless, the DMRs detected in the CHG and CHH contexts are distributed over the first exon and distal intergenic regions, respectively. Hence, this points towards the fine-tuning performed by different methylation contexts in regulating gene expression at different levels (Figure 3C and Figure S3c).

### 3.4. DNA Methylation Levels Modulate Nucleosome Occupancy in Response to Cold Stress

In Arabidopsis, DNA methylation has been found to be more strongly associated with the nucleosome-bound than the flanking DNA [26]. In order to understand how stochastic DNA methylation modulates nucleosome occupancy under normal and cold conditions, we preformed MNase-seq of azadC and Col-0 plants grown at 22 °C and subjected to 4 °C for 24 h, followed by detecting genome-wide nucleosome occupancy using improved nucleosome positioning (iNPS) [76]. Our MNase-seq data demonstrate that Col-0 plants grown at 22 °C display higher number of nucleosome peaks compared to azadC plants for all chromosomes regardless of growth and temperature conditions. We observed that the genome-wide number of nucleosome peaks tend to drop upon cold stress for both azadC plants and Col-0 (Figure S4a). Additionally, our nucleosome distribution over different genomic regions demonstrates that the nucleosome distribution differs between azadC and Col-0 plants, mostly around exons and promoter regions (Figure S4b).

**Figure 4:**
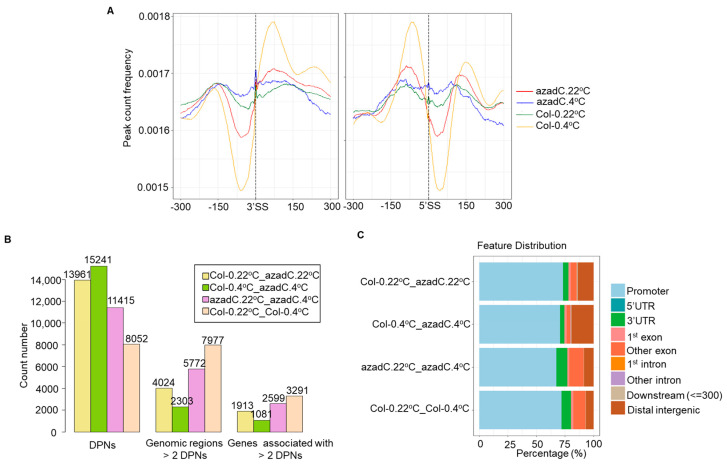
Mnase-Seq data analysis summary. (**A**) Nucleosome profiles across splice sites for azadC and Col-0 at both temperature treatments. The x-axis represents 5′SS and 3′SS alongside 300 bp upstream (−0.3 kb) and downstream (0.3 kb) from the splice sites. The y-axis represents the nucleosome count frequency in different samples. (**B**) Histogram representing the number of differentially positioned nucleosomes (DPNs) detected in each contrast group as well as the number of genomic regions harbouring more than 2 DPNs (genomic regions with >2 DPNs), which have been used to identify the genes (genes) associated with DPNs. The x-axis represents the DPNs and genomic regions and gene count number, and the y-axis represents the count detected in each analysis. (**C**) Percentage of DPN distribution over different genomic features for different contrast groups.

To further understand the relationship between DNA methylation and nucleosome occupancy in regulating the detected AS differences between Col-0 and azadC plants, we profiled nucleosome signals around the 3′SS and 5′SS. Interestingly, while Col-0 plants maintained a clear definition across exons, introns, and exon-intron boundaries, it was evident that the levels of nucleosome occupancy dropped for azadC plants; this trend was even more pronounced upon cold stress treatment (Figure 4A). To confirm that the drop in nucleosome occupancy is not only affecting a subset of the nucleosome detected by iNPS, we applied further stringent criteria. We only plotted nucleosomes with a confidence interval level >95% across splice sites where we obtained similar nucleosome patterns to the ones with a confidence interval level < 95% (Figure 4A and Figure S4c), hence confirming different nucleosome profiles between azadC and Col-0 across splice sites. Surprisingly, when we profiled nucleosomes in −2000/+2000 bp regions flanking the transcription start site (TSS), we could not detect any changes in nucleosome definition between azadC and Col-0 even upon cold stress treatment (Figure S4d). These findings indicate that stochastic changes in DNA methylation are mainly driving the variation in gene expression through modulating nucleosome occupancy around the splice site and probably by modulating RNAPII elongation dynamics and splicing the factor recruitment affecting the splice site selection.

In order to detect differentially positioned nucleosomes (DPNs) and the genes associated with DPNs in different contrast groups, we used DANPOS version 2.1.2 [77] (Figure 4B, Tables S11 and S12). Interestingly, the highest number of DPNs was detected upon azadC treatment alongside cold stress exposure (15241 DPNs). Moreover, we detected more DPNs upon cold stress treatment for azadC plants compared to Col-0 (11,415 and 8052 DPNs; respectively) upon cold stress exposure. By analysing the genomic feature distribution of DPNs detected in different contrast groups, we found that the DPNs detected for azadC and Col-0 plants upon their shift to cold stress are differentially distributed around exons upstream of the promoter regions. (Figure 4C).

Collectively, these data indicate that the stochastic DNA methylation affects nucleosome occupancy mostly around exons located downstream of the promoter regions, which ultimately results in differences in AS profiles detected in our RNA-seq data. Moreover, the role of DNA methylation in regulating nucleosome occupancy is more relevant under cold stress as Arabidopsis plants with differences in DNA methylation displayed the highest number of DPNs upon cold stress.

### 3.5. DNA-Dependant Co-Transcriptional Regulation of Key Metabolic Process under Cold Stress

**Figure 5:**
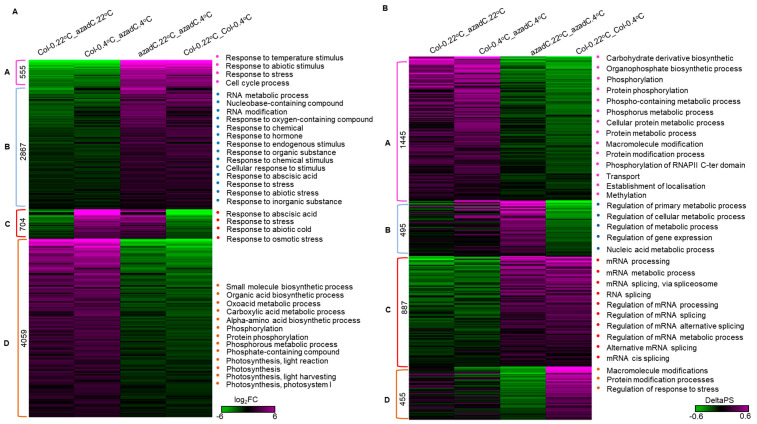
Heatmap showing differentially expressed and alternatively spliced genes k-mean clustering. Clusters of DEGs log_2_FC (**A**) and DAS DeltaPS (**B**) changes detected in at least one contrast group alongside cluster-specific gene ontology terms of enrichment for biological processes.

To further understand the role of DNA methylation in regulating physiological and metabolic pathways, we performed K-means clustering and cluster-specific GO term enrichment of log_2_ Fold Change (log_2_FC) and delta percentage spliced in (deltaPSI) for DE and DAS genes changing in at least one contrast group, respectively. At the gene level, cluster C shows the most intriguing results, as we detected that the temperature-dependant regulation of gene expression is enriched for genes involved in stress (FDR < 2.54 × 10^−22^), cell cycle process (FDR < 2.06 × 10^−16^), and abiotic stimulus (FDR < 4.11 × 10^−16^) (Figure 5, left panel). This regulation seems to be mostly achieved in the cell periphery and the plasma membrane by affecting the microtubule and cytoskeletal-binding activities (Table S4 and Figure S2).

For DAS gene clusters, B and D displayed the differentially regulated pathways between azadC and Col-0 under cold stress. For instance, cluster B for DAS genes demonstrates that temperature dependant changes in DNA methylation are enriched in the regulation of metabolic processes (FDR < 0.003125) terms at different levels, including primary, cellular, nucleic acid, and macromolecule metabolic processes (Figure 5, right panel). Interestingly, we note that azadC plants display an increase in the splicing of genes associated with the aforementioned pathways, while the opposite regulation is being driven by Col-0, hence pointing towards the importance of DNA methylation for stress adaptation through the splicing regulation. Indeed, this is further confirmed by the cluster D, where azadC plants tend to down-regulate the splicing of the stress-responsive genes while Col-0 plants exhibit the opposite trend (FDR < 0.00243) (Table S1d).

To further highlight the role of DNA methylation and nucleosome occupancy in regulating key pathways in Arabidopsis upon cold stress, we performed a Kyoto Encyclopedia of Genes and Genomes (KEGG) enrichment analysis for the genes enriched with DPNs and DMRs detected in our dataset. We found that out of 1088 genes enriched in DPNs for plants with different epigenetic backgrounds under cold stress, 446 were significantly enriched (*p* < 0.05) in plant hormone signal transduction. Conversely, no significant biological pathways were detected for plants with only differences in DNA methylation and grown at normal temperature. Whereas, DMRs detected in the CpG context pathways were mostly enriched in mRNA surveillance. Interestingly, the KEGG enrichment analysis for DMRs in the CHG regions demonstrates an enrichment of oxidative phosphorylation that is enriched only upon cold stress (Figure S5).

Overall, our data indicate that DNA methylation and nucleosome occupancy are essential epigenetic features for co-transcriptional regulation of splicing for genes involved in key metabolic and stress-related pathways, and that context-specific DNA methylation plays a role in regulating the Arabidopsis cold-stress response.

### 3.6. Epigenetic Features Regulate the Directionality of Splicing upon Cold Stress

**Figure 6:**
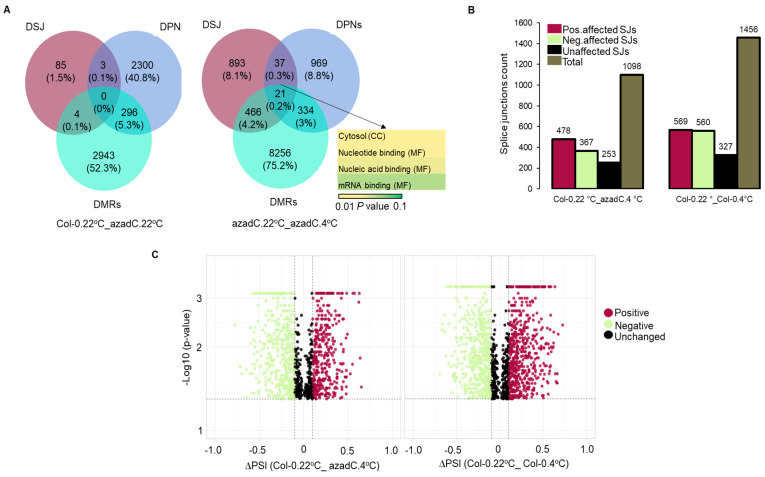
Splicing changes of genes associated with DMRs and DPNs. (**A**) Veen Diagram representing the overlap between genes associated with differential splice junctions (DSJs), differentially positioned nucleosomes (DPNs), and differentially methylated regions (DMRs) in different contrast groups. Gene ontology for molecular functions (MF), and cellular components (CC) has been performed for the overlapping genes, and a corrected *p* value detected for each term is represented as a colour scale. (**B**) Histogram representing the number and directionality of SJs detected for the 21 genes displaying an overlap between DSJs, DPNs, and DMRs. (**C**) Volcano plot representing splicing changes (ΔPSI) of genes with DMRs and DPNs upon cold stress for plants with different (left) and similar (right) genetic background.

To further investigate how DNA methylation and nucleosome occupancy affects SJs’ selection under normal growth conditions and cold stress, we identified genes displaying DMRs, DPNs, and DSJs. We could not detect any genes displaying simultaneous significant changes in splice site selection, nucleosome occupancy, and methylation between azadC and Col-0 grown at normal growth conditions. However, we detected 22 genes exhibiting changes in epigenetic features as well as splicing profiles for azadC exposed to cold stress that were mainly involved in the nucleic-acid-binding activity (Figure 6A). To further understand how changes in stress-induced chromatin remodelling dictate whether SJs in those 22 genes are positively or negatively regulated, we used our SUPPA analysis (Table S3) to extract the AS events that are associated with those genes for the two contrast groups; group (1) plants with different genetic background treated with cold stress and for group (2) Col-0 plants subject to cold stress. Interestingly, for Col-0 plants treated with cold stress, we could identify more (1456 AS events) splicing events compared to group (1) (1098 AS events); the proportion of negatively affected SJs (~38%) was almost equal to the number of positively affected ones (39%). In contrast, the stochastic DNA methylation treatment associated with cold stress in group (1) resulted in ~43 and ~33% positively and negatively affected SJs out of 1098 affected SJs; respectively (Figure 6B). Finally, to investigate whether epigenetic features are involved in affecting the fraction of mRNAs that result from a certain AS event, we have plotted the ΔPSI values of the 22 genes in both groups as above; however, we could not detect a change in the magnitude of AS events (Figure 6C).

Collectively, these data demonstrate that DNA methylation is essential to maintain an exon–intron definition and that a loss of definition around the exon–intron junctions is likely to affect the splice site selection for AS events rather than effecting the fraction of mRNAs that include an exon or specific form of the event.

## 4. Discussion

In eukaryotes, DNA methylation is a conserved epigenetic mark regulating multiple physiological and molecular aspects [83]. Essentially, DNA methylation plays diversified roles in plant responses to different abiotic stresses. For instance, in Arabidopsis, heat stress modulates 5mC through NRPD2, RNA-DEPENDENT RNA POLYMERASE 2 (RDR2), DICER-LIKE 3 (DCL3), and ARGONAUTE 4 (AGO4)-dependent RdDM pathways [69]. Whereas, cold stress decreases 5mC in the promoter of Os03g0610900 to up-regulate its expression, promoting the INDUCER OF CBF EX-PRESSION 1 (ICE1)-mediated cold resistance in rice [84]. Hence, implying a link between transcription and stress-induced epigenetic marks to adapted stress responses. Previously, we also demonstrated in Arabidopsis that there is a coordinated response between cold induced- epigenetic marks and post-transcriptional alternative splicing [22,85].

DNA methylation is not static and can naturally change as a process of adaptation or ageing, leading to rare or stochastic epimutations affecting plant responses to abiotic stresses. Previously, this has been demonstrated where epimutation-related genes are involved directly in drought-responsive pathways [86]. Moreover, cold stress has also been reported to induce somatic stress memory in plants for up to 10 days through modulating histone marks [87]. Cold-induced chromatin modifications were also associated with intergenerational stress memory, resulting in increased homologous recombination frequency [88]. Subsequently, stochastic DNA methylation or epimutations affecting cold responsive genes can be inherited through somatic or meiotic stress memory, thereby affecting direct or subsequent progeny stress responses.

Our hcDMRs analysis demonstrate that azadC plants displayed a genome-wide increase in hypomethylated regions, which is in line with previous studies from Arabidopsis [89] (Figure 3B). These DMRs were mainly distributed over the promoter region and exons up-stream of the promoter regions (Figure 3C), thereby affecting transcription and AS. To explore the effect of DNA hypomethylation, we interrogated our RNA-Seq data to reveal that despite an increase in the number of genes upon cold stress treatment for azadC plants compared to Col-0, the up-regulated DAS genes number remained higher for Col-0 plants, which is a key defence mechanism in the plant cold-stress response [12] (Figure 2A). Indeed, we could clearly detect from our PGSEA that for azadC plants, the AS of defence and stress response-related genes were repressed compared to Col-0 plants (Figure 2B and Figure 5B). Moreover, our cluster analysis demonstrated that azadC plants, compared to Col-0 plants under cold stress, display strong splicing repression of splicing factors and an increase in RNAPII tail C-terminal domain phosphorylation (Figure 2B and Figure 5A,B). Hence, this explains the decreased splicing and increased number of up-regulated genes in azadC plants. The decrease splicing in this latter was also confirmed by the decrease in positively regulated SJs for azadC plants compared to Col-0 upon cold stress treatment. Interestingly, we also detected that azadC treated plants display decreased splicing of protein modification-related genes when compared to Col-0 (Figure 5B), which could subsequently result in decreased protein diversity.

DNA methylation and nucleosome occupancy can affect co-transcriptional splicing as previously reported in human and plants [22,30,85]. We found that stochastic DNA methylation affects nucleosome occupancy and levels more strongly upon cold stress treatment, where azadC treatment coupled with cold stress resulted in 15,241 DPNs alongside the lowest levels of positioned nucleosome (Figure 4A,B). Interestingly, DPNs’ global distribution were similar to DMRs’, whereby DPNs resulting from the azadC treatment were also located around promoters and exons upstream the promoter regions to affect transcription and AS, respectively. From this perspective, we propose that stochastic DNA demethylation as a result of 5-azadC treatment is associated with nucleosome remodelling to affect AS events and ratios upon cold abiotic stress. Our results are in line with previous studies in Arabidopsis, where in four populations of the latter, DNA demethylation with 5-azadC resulted in a 30–78% increase in freezing tolerance, a trend that was also observed in drm2 mutants [90].

Taken together, both DNA methylation and nucleosome occupancy are essential for exon-intron definition (Figure 4A). Subsequently, this affects SF recruitment and cold-induced AS regulation. Our results demonstrate that stochastic DNA hypomethylation results in decreased RNA-binding activity, which potentially reflects on RNA–protein interactions and SF recruitment, which are essential for chromatin remodelling and AS regulation (Figure S2, DAS MF). Interestingly, this is further confirmed by our results, which demonstrate that the 5-azadC treatment results in 22 genes displaying DPNs, negatively affected DSJs, and DMRs enriched mostly in the mRNA and nucleotide binding (Figure 6A,B).

Stochastic changes in DNA methylation can regulate multiple aspects of plant cellular physiology in response to cold stress. For instance, we detected 1594 genes displaying DMRs upon 5-azadC treatment in the CHG context-regulating mRNA surveillance pathway and basal transcription factors, and 23 genes regulating amino acid biosynthesis and metabolism as well as protein post-translational modifications. This was in line with previous reports proposing context-specific stress-induced transgenerational DNA methylation variation in dandelions [91]. Moreover, azadC plants treated with cold stress displayed a differential nucleosome occupancy compared to Col-0 in 446 genes related to hormone signal transduction (Figure S5).

Overall, our data show that similar to stable changes in DNA methylation [22], rare and non-static epimutations can affect cold-stress adaptation through affecting mRNA-binding affinity to SFs, chromatin remodellers, mRNA surveillance pathways, and thereby, AS splicing.

It remains to be determined whether such a regulation can be mitotically or meiotically inherited, and whether transgenerational inheritance can be associated with phenotypic changes tagged by specific chromatin marks. If further investigated, this could help manipulate somatic and transgenerational epigenetic memory to enhance the crops’ response and tolerance to cold stress.

## 5. Conclusions

DNA methylation is not static and can be affected by environmental variation and epimutations. We endeavoured to understand how stochastic DNA methylation affects Arabidopsis cold stress response and what the molecular and physiological aspects targeted are by such a regulation. Here, we report that stochastic DNA methylation and nucleosome occupancy levels are associated with distinct gene expression and splicing profiles in response to cold stress. These distinct profiles affected genes involved in cellular metabolic processes and stress regulation. We also demonstrate that this DNA-dependent cold stress response was associated with a decrease in amino acid synthesis and down-regulate post-translational protein metabolic processes and modifications, which could be a direct consequence of the dysfunction of alternative mRNA splicing.

## Data Availability

All RNA-Seq, WGBS, and MNase-Seq raw data generated in this work are publicly accessible through NCBI-SRA accessions PRJNA592356.

## Supplementary Materials

The following supporting information can be downloaded at: https://www.mdpi.com/article/10.3390/plants11091105/s1, Figure S1: Differentially expressed and alternatively spliced genes analysis, Figure S2: Parametric gene set enrichment analysis defining molecular functions, Figure S3: Bisulphite-Seq profiles and genomic distribution in the CHG and CHH contexts, Figure S4: Nucleosome genome-wide distribu-tion and profiles of Col-0 and azadC plants grown at 22 °C and 4 °C, Figure S5: KEGG dot plot enriched GO terms. Table S1: Summary of differentially expressed (DE) and alternatively spliced (DAS) genes identified by 3D RNA-Seq pipeline. Table S2: Preranked gene set enrichment analysis for differentially expressed and differentially alternatively spliced genes. Table S3: Significant (*p* value ≤ 0.05) differential splicing events for local alternative splicing events (ΔPSI value) detected by SUPPA in different contrast groups. Table S4: Gene ontology enrichment analysis for each cluster for differentially expressed and alternatively spliced genes. Table S5: High confidence differentially methylated regions (hcDMRs) (Fisher’s exact test *p* value ≤ 0.01) for aza-Dc samples at 22 °C in CpG context. Table S6: High confidence differentially methylated regions (hcDMRs) (Fisher’s exact test *p* value ≤ 0.01) for aza-Dc samples at 22 °C in CHG context. Table S7: High confidence differentially methylated regions (hcDMRs) (Fisher’s exact test *p* value ≤ 0.01) for aza-dc samples at 22 °C in CHH context. Table S8: High confidence differentially methylated regions (hcDMRs) (Fisher’s exact test *p* value ≤ 0.01) for aza-dc samples at 4 °C in CpG context. Table S9: High confidence differentially methylated regions (hcDMRs) (Fisher’s exact test *p* value ≤ 0.01) for aza-dc samples at 4 °C in CHG context. Table S10: High confidence differentially methylated regions (hcDMRs) (Fisher’s exact test *p* value ≤ 0.01) for aza-dC samples at 4 °C in CHH context. Table S11: Association of significantly differentially positioned nucleosomes in windows that have ≥2 differentially positioned nucleo-somes with nearby genes. Table S12: Count of differentially positioned nucleosomes within 2000 bp sliding windows with a step size of 500 bp across Arabidopsis genome.

## References

[1] Yu H., Tian C., Yu Y., Jiao Y. (2016). Transcriptome Survey of the Contribution of Alternative Splicing to Proteome Diversity in Arabidopsis thaliana. Mol. Plant.

[2] Liu J., Feng L., Li J., He Z. (2015). Genetic and epigenetic control of plant heat responses. Front. Plant Sci..

[3] Syed N.H., Kalyna M., Marquez Y., Barta A., Brown J.W.S. (2012). Alternative splicing in plants—Coming of age. Trends Plant Sci..

[4] Reddy A.S.N., Marquez Y., Kalyna M., Barta A. (2013). Complexity of the alternative splicing landscape in plants. Plant Cell.

[5] Jabre I., Reddy A.S.N., Kalyna M., Chaudhary S., Khokhar W., Byrne L.J., Wilson C.M., Syed N.H. (2019). Does co-transcriptional regulation of alternative splicing mediate plant stress responses?. Nucleic Acids Res..

[6] Marquez Y., Brown J.W.S., Simpson C., Barta A., Kalyna M. (2012). Transcriptome survey reveals increased complexity of the alternative splicing landscape in Arabidopsis. Genome Res..

[7] Zhang G., Guo G., Hu X., Zhang Y., Li Q., Li R., Zhuang R., Lu Z., He Z., Fang X. (2010). Deep RNA sequencing at single base-pair resolution reveals high complexity of the rice transcriptome. Genome Res..

[8] Chamala S., Feng G., Chavarro C., Barbazuk W.B. (2015). Genome-Wide Identification of Evolutionarily Conserved Alternative Splicing Events in Flowering Plants. Front. Bioeng. Biotechnol..

[9] Filichkin S.A., Hamilton M., Dharmawardhana P.D., Singh S.K., Sullivan C., Ben-Hur A., Reddy A.S., Jaiswal P. (2018). Abiotic stresses modulate landscape of poplar transcriptome via alternative splicing, differential intron retention, and isoform ratio switching. Front. Plant Sci..

[10] Mastrangelo A.M., Marone D., Laidò G., De Leonardis A.M., De Vita P. (2012). Alternative splicing: Enhancing ability to cope with stress via transcriptome plasticity. Plant Sci..

[11] Chaudhary S., Jabre I., Reddy A.S.N., Staiger D., Syed N.H. (2019). Perspective on Alternative Splicing and Proteome Complexity in Plants. Trends Plant Sci..

[12] Calixto C.P.G., Guo W., James A.B., Tzioutziou N.A., Entizne J.C., Panter P.E., Knight H., Nimmo H., Zhang R., Brown J.W.S. (2018). Rapid and dynamic alternative splicing impacts the Arabidopsis cold response transcriptome. Plant Cell.

[13] Streitner C., Simpson C.G., Shaw P., Danisman S., Brown J.W.S., Staiger D. (2013). Small changes in ambient temperature affect alternative splicing in Arabidopsis thaliana. Plant Signal. Behav..

[14] James A.B., Syed N.H., Bordage S., Marshall J., Nimmo G.A., Jenkins G.I., Herzyk P., Brown J.W.S., Nimmo H.G. (2012). Alternative Splicing Mediates Responses of the Arabidopsis Circadian Clock to Temperature Changes. Plant Cell.

[15] Filichkin S.A., Cumbie J.S., Dharmawardhana P., Jaiswal P., Chang J.H., Palusa S.G., Reddy A.S.N., Megraw M., Mockler T.C. (2015). Environmental stresses modulate abundance and timing of alternatively spliced circadian transcripts in Arabidopsis. Mol. Plant.

[16] Ding Y., Tang Y., Kwok C.K., Zhang Y., Bevilacqua P.C., Assmann S.M. (2014). In vivo genome-wide profiling of RNA secondary structure reveals novel regulatory features. Nature.

[17] Shen H., Kan J.L.C., Green M.R. (2004). Arginine-Serine-Rich Domains Bound at Splicing Enhancers Contact the Branchpoint to Promote Prespliceosome Assembly. Mol. Cell.

[18] Hajheidari M., Koncz C., Eick D. (2013). Emerging roles for RNA polymerase II CTD in Arabidopsis. Trends Plant Sci..

[19] Lenasi T., Barboric M. (2010). P-TEFb stimulates transcription elongation and pre-mRNA splicing through multilateral mechanisms. RNA Biol..

[20] Hirose Y., Manley J.L. (2000). RNA polymerase II and the integration of nuclear events. Genes Dev..

[21] Gasch A., Wiesner S., Martin-Malpartida P., Ramirez-Espain X., Ruiz L., Macias M.J. (2006). The structure of Prp40 FF1 domain and its interaction with the crn-TPR1 motif of Clf1 gives a new insight into the binding mode of FF domains. J. Biol. Chem..

[22] Chaudhary S., Jabre I., Syed N.H. (2021). Epigenetic differences in an identical genetic background modulate alternative splicing in A. thaliana. Genomics.

[23] Ehrlich M., Gama-Sosa M.A., Huang L.H., Midgett R.M., Kuo K.C., Mccune R.A., Gehrke C. (1982). Amount and distribution of 5-methylcytosine in human DNA from different types of tissues or cells. Nucleic Acids Res..

[24] Cokus S.J., Feng S., Zhang X., Chen Z., Merriman B., Haudenschild C.D., Pradhan S., Nelson S.F., Pellegrini M., Jacobsen S.E. (2008). Shotgun bisulphite sequencing of the Arabidopsis genome reveals DNA methylation patterning. Nature.

[25] Lister R., O’Malley R.C., Tonti-Filippini J., Gregory B.D., Berry C.C., Millar A.H., Ecker J.R. (2008). Highly Integrated Single-Base Resolution Maps of the Epigenome in Arabidopsis. Cell.

[26] Chodavarapu R.K., Feng S., Bernatavichute Y.V., Chen P.-Y.Y., Stroud H., Yu Y., Hetzel J.A., Kuo F., Kim J., Cokus S.J. (2010). Relationship between nucleosome positioning and DNA methylation. Nature.

[27] Schwartz S., Meshorer E., Ast G. (2009). Chromatin organization marks exon-intron structure. Nat. Struct. Mol. Biol..

[28] Mavrich T.N., Jiang C., Ioshikhes I.P., Li X., Venters B.J., Zanton S.J., Tomsho L.P., Qi J., Glaser R.L., Schuster S.C. (2008). Nucleosome organization in the Drosophila genome. Nature.

[29] Liu M.-J., Seddon A.E., Tsai Z.T.-Y., Major I.T., Floer M., Howe G.A., Shiu S.-H. (2015). Determinants of nucleosome positioning and their influence on plant gene expression. Genome Res..

[30] Gelfman S., Cohen N., Yearim A., Ast G. (2013). DNA-methylation effect on cotranscriptional splicing is dependent on GC architecture of the exon-intron structure. Genome Res..

[31] Tilgner H., Nikolaou C., Althammer S., Sammeth M., Beato M., Valcárcel J., Guigó R. (2009). Nucleosome positioning as a determinant of exon recognition. Nat. Struct. Mol. Biol..

[32] Chen W., Luo L., Zhang L. (2010). The organization of nucleosomes around splice sites. Nucleic Acids Res..

[33] Nahkuri S., Taft R.J., Mattick J.S. (2009). Nucleosomes are preferentially positioned at exons in somatic and sperm cells. Cell Cycle.

[34] Huff J.T., Zilberman D. (2014). Dnmt1-independent CG methylation contributes to nucleosome positioning in diverse eukaryotes. Cell.

[35] Alexander R.D., Innocente S.A., Barrass J.D., Beggs J.D. (2010). Splicing-Dependent RNA polymerase pausing in yeast. Mol. Cell.

[36] Ullah F., Hamilton M., Reddy A.S.N., Ben-Hur A. (2018). Exploring the relationship between intron retention and chromatin accessibility in plants. BMC Genom..

[37] Zhu J., Liu M., Liu X., Dong Z. (2018). RNA polymerase II activity revealed by GRO-seq and pNET-seq in Arabidopsis. Nat. Plants.

[38] Garg R., Narayana Chevala V., Shankar R., Jain M. (2015). Divergent DNA methylation patterns associated with gene expression in rice cultivars with contrasting drought and salinity stress response. Sci. Rep..

[39] Secco D., Wang C., Shou H., Schultz M.D., Chiarenza S., Nussaume L., Ecker J.R., Whelan J., Lister R. (2015). Stress induced gene expression drives transient DNA methylation changes at adjacent repetitive elements. Elife.

[40] Lu X., Wang X., Chen X., Shu N., Wang J., Wang D., Wang S., Fan W., Guo L., Guo X. (2017). Single-base resolution methylomes of upland cotton (Gossypium hirsutum L.) reveal epigenome modifications in response to drought stress. BMC Genom..

[41] Dowen R.H., Pelizzola M., Schmitz R.J., Lister R., Dowen J.M., Nery J.R., Dixon J.E., Ecker J.R. (2012). Widespread dynamic DNA methylation in response to biotic stress. Proc. Natl. Acad. Sci. USA.

[42] Steward N., Ito M., Yamaguchi Y., Koizumi N., Sano H. (2002). Periodic DNA methylation in maize nucleosomes and demethylation by environmental stress. J. Biol. Chem..

[43] Robertson K.D. (2005). DNA methylation and human disease. Nat. Rev. Genet..

[44] Eden A., Gaudet F., Waghmare A., Jaenisch R. (2003). Chromosomal instability and tumors promoted by DNA hypomethylation. Science.

[45] Christman J. (2002). 5-Azacytidine and 5-aza-2’-deoxycytidine as inhibitors of DNA methylation: Mechanistic studies and their implications for cancer therapy. Oncogene.

[46] Sano H., Kamada I., Youssefian S., Katsumi M., Wabiko H. (1990). A single treatment of rice seedlings with 5-azacytidine induces heritable dwarfism and undermethylation of genomic DNA. MGG Mol. Gen. Genet..

[47] Kumpatla S.P., Teng W., Buchholz W.G., Hall T.C. (1997). Epigenetic Transcriptional Silencing and 5-Azacytidine-Mediated Reactivation of a Complex Transgene in Rice. Plant Physiol..

[48] Fieldes M.A. (1994). Heritable effects of 5-azacytidine treatments on the growth and development of flax (Linum usitatissimum) genotrophs and genotypes. Genome.

[49] Vyskot B., Koukalová B., Kovařík A., Sachambula L., Reynolds D., Bezděk M. (1995). Meiotic transmission of a hypomethylated repetitive DNA family in tobacco. Theor. Appl. Genet..

[50] King G.J. (1995). Morphological development in Brassica oleracea is modulated by in vivo treatment with 5-azacytidine. J. Hortic. Sci..

[51] Janoušek B., Široký J., Vyskot B. (1996). Epigenetic control of sexual phenotype in a dioecious plant, Melandrium album. Mol. Gen. Genet..

[52] Amado L., Abranches R., Neves N., Viegas W. (1997). Development-dependent inheritance of 5-azacytidine-induced epimutations in triticale: Analysis of rDNA expression patterns. Chromosom. Res..

[53] Burn J.E., Bagnall D.J., Metzger J.D., Dennis E.S., Peacock W.J. (1993). DNA methylation, vernalization, and the initiation of flowering. Proc. Natl. Acad. Sci. USA.

[54] Xu J., Tanino K.K., Horner K.N., Robinson S.J. (2016). Quantitative trait variation is revealed in a novel hypomethylated population of woodland strawberry (Fragaria vesca). BMC Plant Biol..

[55] Marfil C.F., Asurmendi S., Masuelli R.W. (2012). Changes in micro RNA expression in a wild tuber-bearing Solanum species induced by 5-Azacytidine treatment. Plant Cell Rep..

[56] Thomashow M.F. (2010). Molecular Basis of Plant Cold Acclimation: Insights Gained from Studying the CBF Cold Response Pathway. Plant Physiol..

[57] Knight M.R., Knight H. (2012). Low-temperature perception leading to gene expression and cold tolerance in higher plants. New Phytol..

[58] Barrero-Gil J., Salinas J. (2013). Post-translational regulation of cold acclimation response. Plant Sci..

[59] Palusa S.G., Ali G.S., Reddy A.S.N. (2007). Alternative splicing of pre-mRNAs of Arabidopsis serine/arginine-rich proteins: Regulation by hormones and stresses. Plant J..

[60] Pan Y., Wang W., Zhao X., Zhu L., Fu B., Li Z. (2011). DNA methylation alterations of rice in response to cold stress. Plant Omics.

[61] McClung C.R., Davis S.J. (2010). Ambient thermometers in plants: From physiological outputs towards mechanisms of thermal sensing. Curr. Biol..

[62] Kumar S.V., Wigge P.A. (2010). H2A.Z-Containing Nucleosomes Mediate the Thermosensory Response in Arabidopsis. Cell.

[63] Roy D., Paul A., Roy A., Ghosh R., Ganguly P., Chaudhuri S. (2014). Differential acetylation of histone H3 at the regulatory region of OsDREB1b promoter facilitates chromatin remodelling and transcription activation during cold stress. PLoS ONE.

[64] Bolger A.M., Lohse M., Usadel B. (2014). Trimmomatic: A flexible trimmer for Illumina sequence data. Bioinformatics.

[65] Patro R., Duggal G., Love M.I., Irizarry R.A., Kingsford C. (2017). Salmon provides fast and bias-aware quantification of transcript expression. Nat. Methods.

[66] Zhang R., Calixto C.P.G., Marquez Y., Venhuizen P., Tzioutziou N.A., Guo W., Spensley M., Entizne J.C., Lewandowska D., Have S.T. (2017). A high quality Arabidopsis transcriptome for accurate transcript-level analysis of alternative splicing. Nucleic Acids Res..

[67] Soneson C., Love M.I., Robinson M.D. (2015). Differential analyses for RNA-seq: Transcript-level estimates improve gene-level inferences. F1000Research.

[68] Bullard J.H., Purdom E., Hansen K.D., Dudoit S. (2010). Evaluation of statistical methods for normalization and differential expression in mRNA-Seq experiments. BMC Bioinform..

[69] Robinson M.D., McCarthy D.J., Smyth G.K. (2009). edgeR: A Bioconductor package for differential expression analysis of digital gene expression data. Bioinformatics.

[70] Ritchie M.E., Phipson B., Wu D., Hu Y., Law C.W., Shi W., Smyth G.K. (2015). Limma powers differential expression analyses for RNA-sequencing and microarray studies. Nucleic Acids Res..

[71] Huang D.W., Sherman B.T., Lempicki R.A. (2009). Systematic and integrative analysis of large gene lists using DAVID bioinformatics resources. Nat. Protoc..

[72] Huang D.W., Sherman B.T., Lempicki R.A. (2009). Bioinformatics enrichment tools: Paths toward the comprehensive functional analysis of large gene lists. Nucleic Acids Res..

[73] Trincado J.L., Entizne J.C., Hysenaj G., Singh B., Skalic M., Elliott D.J., Eyras E. (2018). SUPPA2: Fast, accurate, and uncertainty-aware differential splicing analysis across multiple conditions. Genome Biol..

[74] Alamancos G.P., Pagès A., Trincado J.L., Bellora N., Eyras E. (2015). Leveraging transcript quantification for fast computation of alternative splicing profiles. RNA.

[75] Langmead B., Trapnell C., Pop M., Salzberg S.L. (2009). Ultrafast and memory-efficient alignment of short DNA sequences to the human genome. Genome Biol..

[76] Chen W., Liu Y., Zhu S., Green C.D., Wei G., Han J.D.J. (2014). Improved nucleosome-positioning algorithm iNPS for accurate nucleosome positioning from sequencing data. Nat. Commun..

[77] Chen K., Xi Y., Pan X., Li Z., Kaestner K., Tyler J., Dent S., He X., Li W. (2013). DANPOS: Dynamic analysis of nucleosome position and occupancy by sequencing. Genome Res..

[78] Krueger F., Andrews S.R. (2011). Bismark: A flexible aligner and methylation caller for Bisulfite-Seq applications. Bioinformatics.

[79] Akalin A., Kormaksson M., Li S., Garrett-Bakelman F.E., Figueroa M.E., Melnick A., Mason C.E. (2012). MethylKit: A comprehensive R package for the analysis of genome-wide DNA methylation profiles. Genome Biol..

[80] Griffin P.T., Niederhuth C.E., Schmitz R.J. (2016). A comparative analysis of 5-azacytidine-and zebularine-induced DNA demethylation. G3 Genes Genomes Genet..

[81] Wang X., Hu L., Wang X., Li N., Xu C., Gong L., Liu B. (2016). DNA Methylation Affects Gene Alternative Splicing in Plants: An Example from Rice. Mol. Plant.

[82] Zhang Y., Harris C.J., Liu Q., Liu W., Ausin I., Long Y., Xiao L., Feng L., Chen X., Xie Y. (2018). Large-scale comparative epigenomics reveals hierarchical regulation of non-CG methylation in Arabidopsis. Proc. Natl. Acad. Sci. USA.

[83] Law J., Jacobsen S. (2010). Establishing, maintaining and modifying DNA methylation patterns in plants and animals. Nat. Rev. Genet..

[84] Guo H., Wu T., Li S., He Q., Yang Z., Zhang W. (2019). The methylation patterns and transcriptional responses to chilling stress at the seedling stage in rice. Int. J. Mol. Sci..

[85] Jabre I., Chaudhary S., Guo W., Kalyna M., Reddy A.S.N., Chen W., Zhang R., Wilson C., Syed N.H. (2021). Differential nucleosome occupancy modulates alternative splicing in Arabidopsis thaliana. New Phytol..

[86] Zheng X., Chen L., Xia H., Wei H., Lou Q., Li M., Li T., Luo L. (2017). Transgenerational epimutations induced by multi-generation drought imposition mediate rice plant’s adaptation to drought condition. Sci. Rep..

[87] Bäurle I., Trindade I. (2020). Chromatin regulation of somatic abiotic stress memory. J. Exp. Bot..

[88] Boyko A., Blevins T., Yao Y., Golubov A., Bilichak A., Ilnytskyy Y., Hollander J., Meins F., Kovalchuk I. (2010). Transgenerational adaptation of Arabidopsis to stress requires DNA methylation and the function of Dicer-like proteins. PLoS ONE.

[89] Xie H., Sun Y., Cheng B., Xue S., Cheng D., Liu L., Meng L., Qiang S. (2019). Variation in ICE1 methylation primarily determines phenotypic variation in freezing tolerance in Arabidopsis thaliana. Plant Cell Physiol..

[90] Chang S., Pikaard C.S. (2005). Transcript profiling in Arabidopsis reveals complex responses to global inhibition of DNA methylation and histone deacetylation. J. Biol. Chem..

[91] Preite V., Oplaat C., Biere A., Kirschner J., Van Der Putten W.H., Verhoeven K.J.F. (2018). Increased transgenerational epigenetic variation, but not predictable epigenetic variants, after environmental exposure in two apomictic dandelion lineages. Ecol. Evol..

