# Supplementary material for: Stochastic Variation in DNA Methylation Modulates Nucleosome Occupancy and Alternative Splicing in Arabidopsis thaliana
Source: Plants (Basel). 2022 Apr 19;11(9):1105. doi: 10.3390/plants11091105 (PMC9101026; doi:10.3390/plants11091105)
Supplement: Supplementary file 1 [file plants-11-01105-s001.zip › Supplementary figures legends.pdf]

Supplementary figures legends

Figure S1. Differentially expressed and alternatively spliced genes analysis. (a) Principal components analysis of the gene (upper panel) and transcript (lower panel) levels. Dimensions (Dim) 1 and 2 are temperature and 5-azadC treatment variance; respectively. (b). Veen diagram representing the overlap between differentially expressed (DE) and differentially alternatively spliced (DAS) genes in each contrast group. (c) Veen diagram representing the overlap between differentially expressed genes (upper lane) as well as differentially alternatively spliced genes (lower lane) detected for the different contrast group. The scale represents the gene count number.

Figure S2. Parametric gene set enrichment analysis defining molecular functions (a) and cellular components (b) gene ontology terms enriched in each contrast group for differentially expressed (DE) and differentially alternatively spliced (DAS) genes. Red and blue indicates activated and suppressed pathways, respectively.

Figure S3. Bisulphite-Seq profiles and genomic distribution in the CHG and CHH contexts. DNA methylation profiles across splice sites for azadC plants at 22ºC (top panel) and 4ºC (lower panel) . The x-axis represents 5'SS and 3'SS alongside 300 bp upstream (-0.3kb) and downstream (0.3kb) the splice sites. The y-axis represents the DNA methylation count frequency with a confidence interval (0.95) estimated by bootstrap method. (b) Percentage of DMRs distribution over different genomic features for different contrast groups in the CHG (left panel) and CHH (right panel) contexts.

Figure S4. Nucleosome genome-wide distribution and profiles of Col-0 and azadC plants grown at 22°C and 4°C. (a) Nucleosome distribution over Arabidopsis 5 chromosomes (Chr) in each sample. (b) Nucleosome distribution over different genomic features for different samples (c) Nucleosome profiles around splice sites with a confidence interval (0.95) estimated by bootstrap method for azadC and Col-0 at both temperature treatments. The x-axis represents 5'SS and 3'SS alongside 300 bp upstream (-0.3kb) and downstream (0.3kb) the splice sites. The y-axis represents the nucleosome count frequency in different samples. (d) Nucleosome peaks detected by iNPS at the transcription start site (TSS) for azadC and Col-0 at both temperature treatments. The x-axis represents the distribution of nucleosome density in -2000/+2000 bp regions flanking the TSS. The y-axis represents the nucleosome count frequency in different samples.

Figure S5. KEGG dot plot enriched GO terms. Functional profiles of DPNs (a) and DMRs in both CpG and CHG contexts (b). The size of the dots represent the number of genes in DPNs or DMRs list associated with the GO term and the color of the dots represent the P-adjusted values (BH).
