# Supplementary figures and images for: Stochastic Variation in DNA Methylation Modulates Nucleosome Occupancy and Alternative Splicing in Arabidopsis thaliana
Source: Plants (Basel). 2022 Apr 19;11(9):1105. doi: 10.3390/plants11091105 (PMC9101026; doi:10.3390/plants11091105)

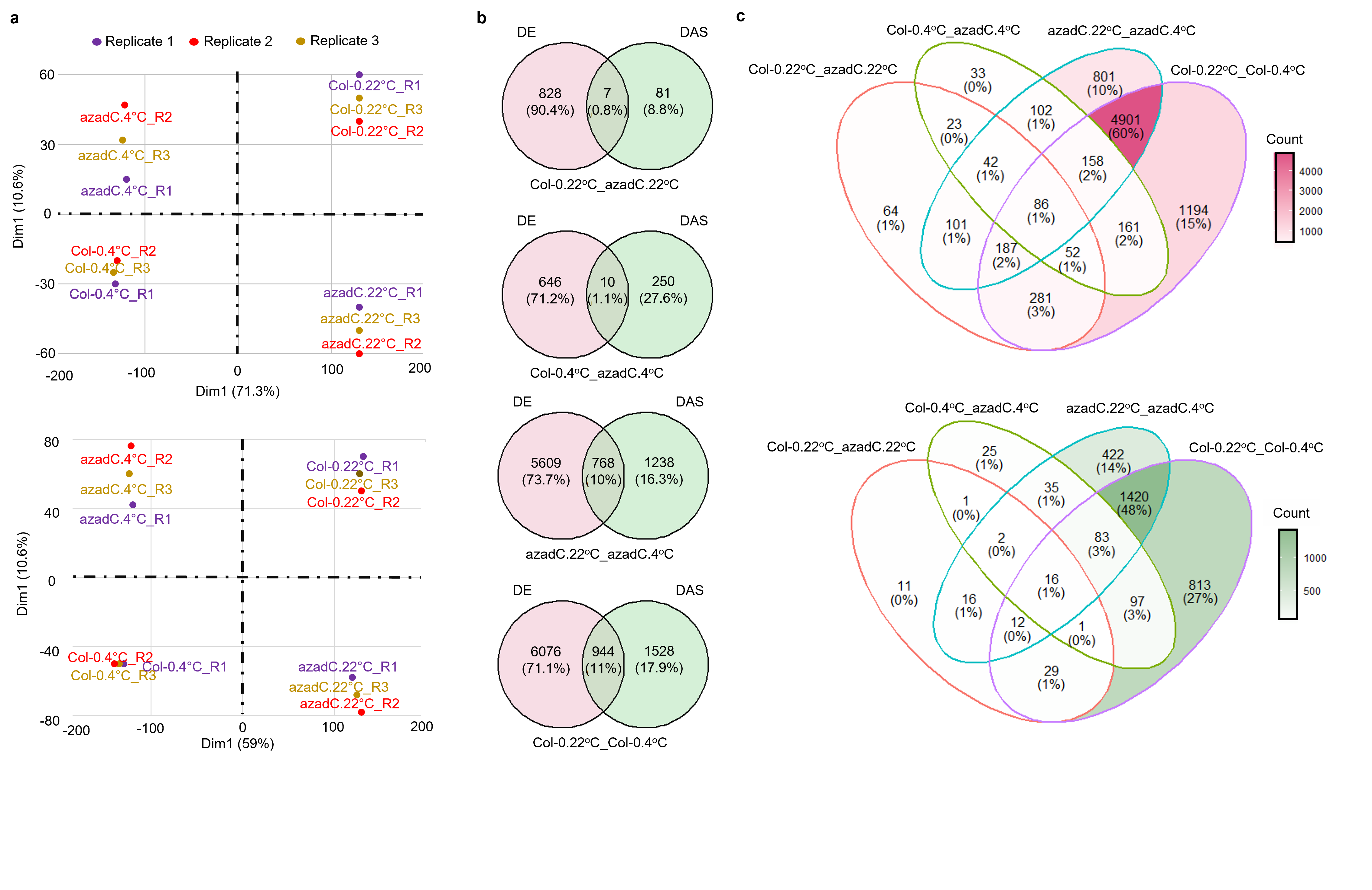

Supplement: Supplementary file 1 [file plants-11-01105-s001.zip › Figure S1.png]

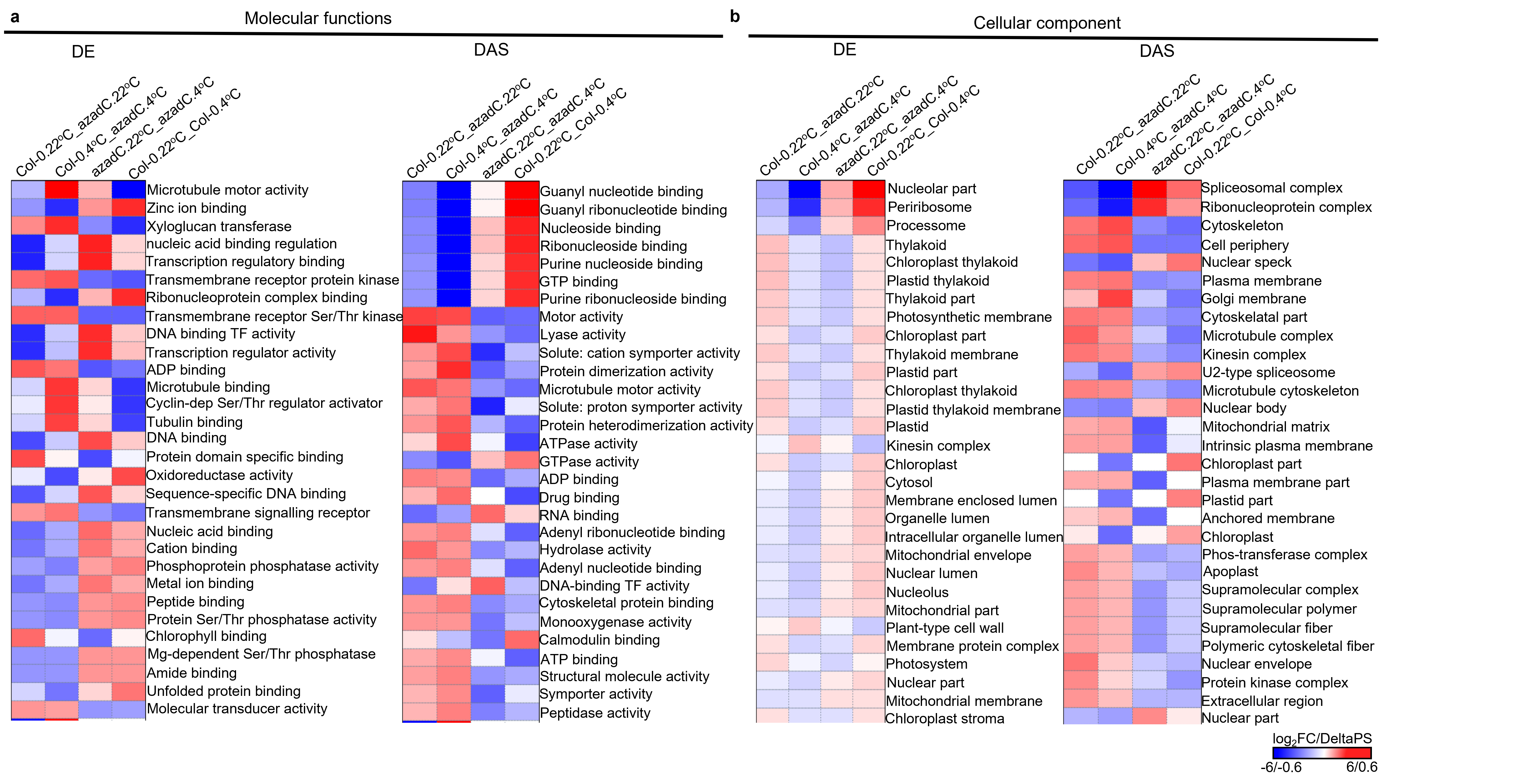

Supplement: Supplementary file 1 [file plants-11-01105-s001.zip › Figure S2.png]

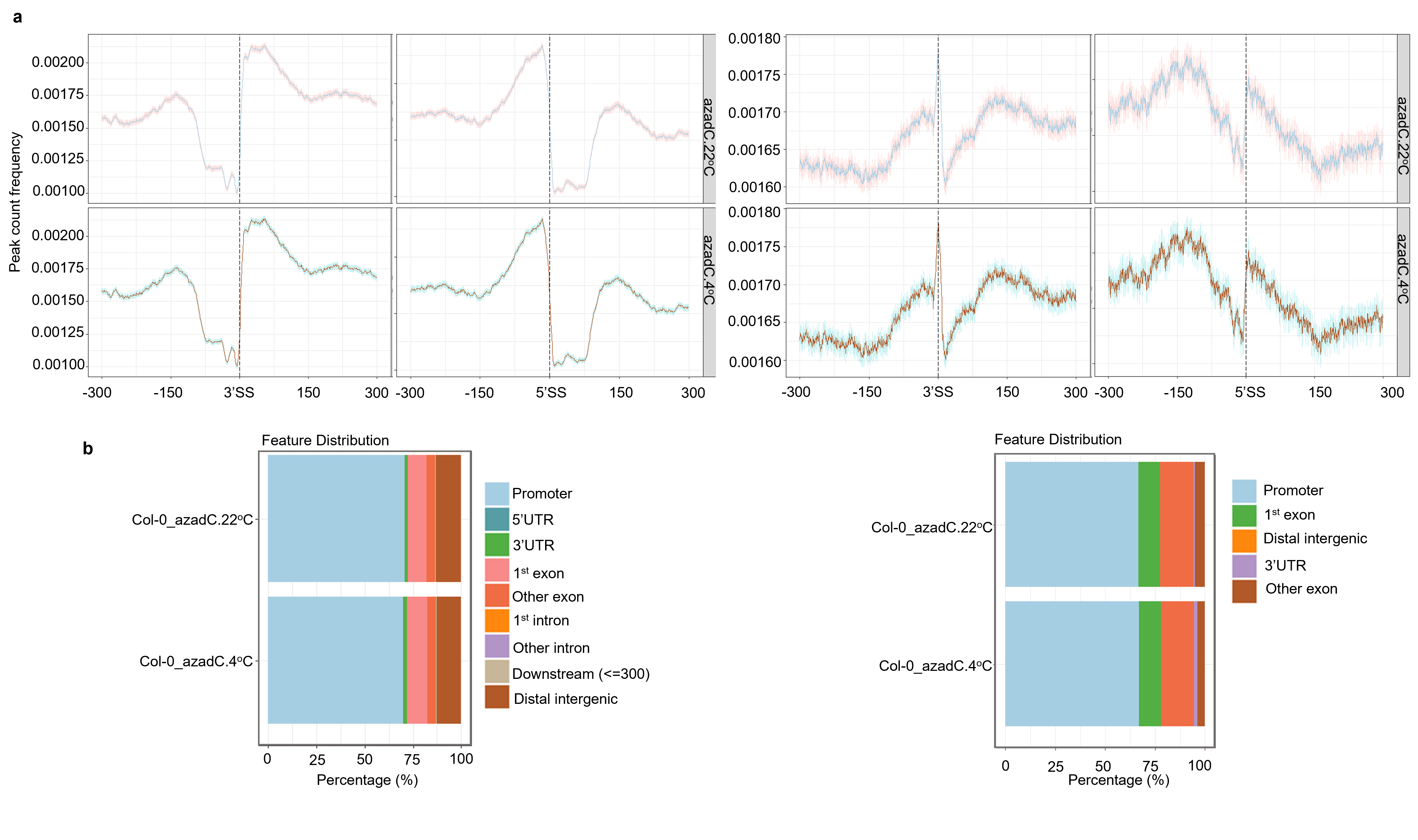

Supplement: Supplementary file 1 [file plants-11-01105-s001.zip › Figure S3.png]

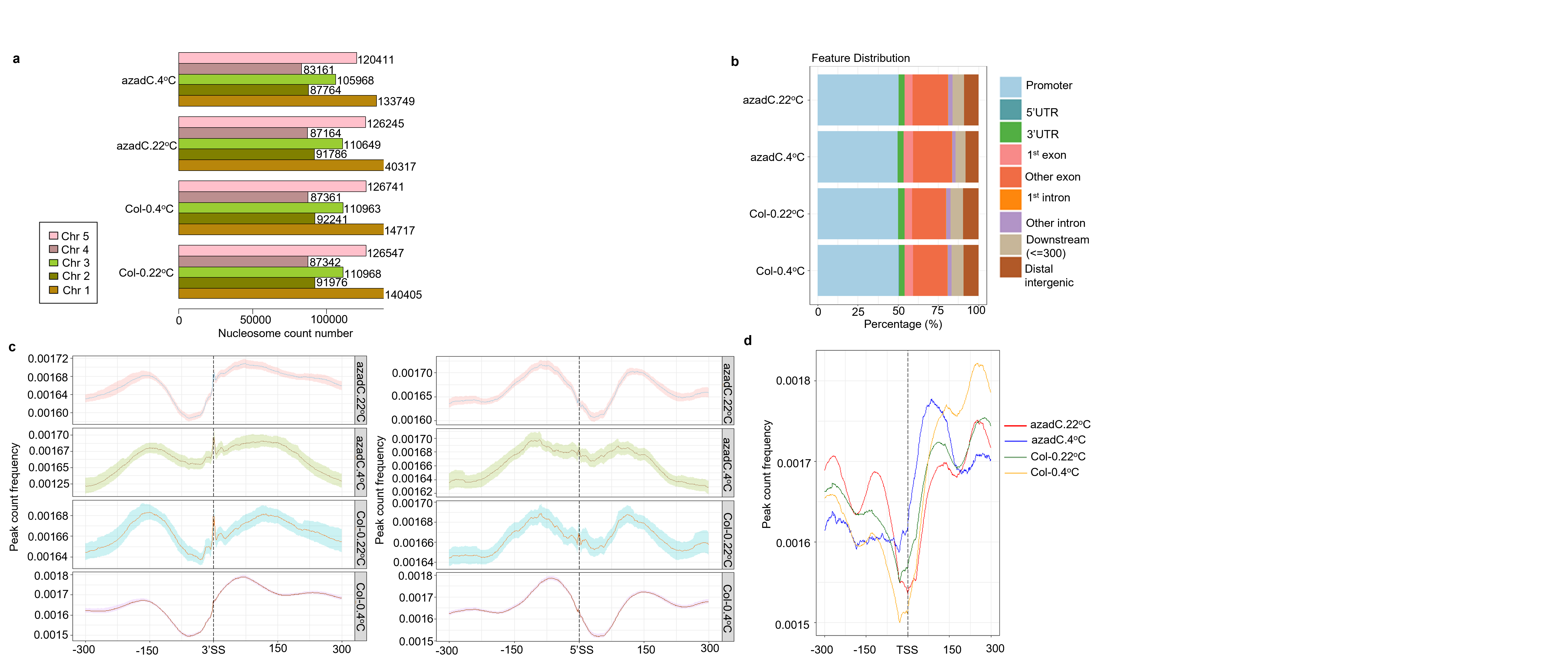

Supplement: Supplementary file 1 [file plants-11-01105-s001.zip › Figure S4.png]

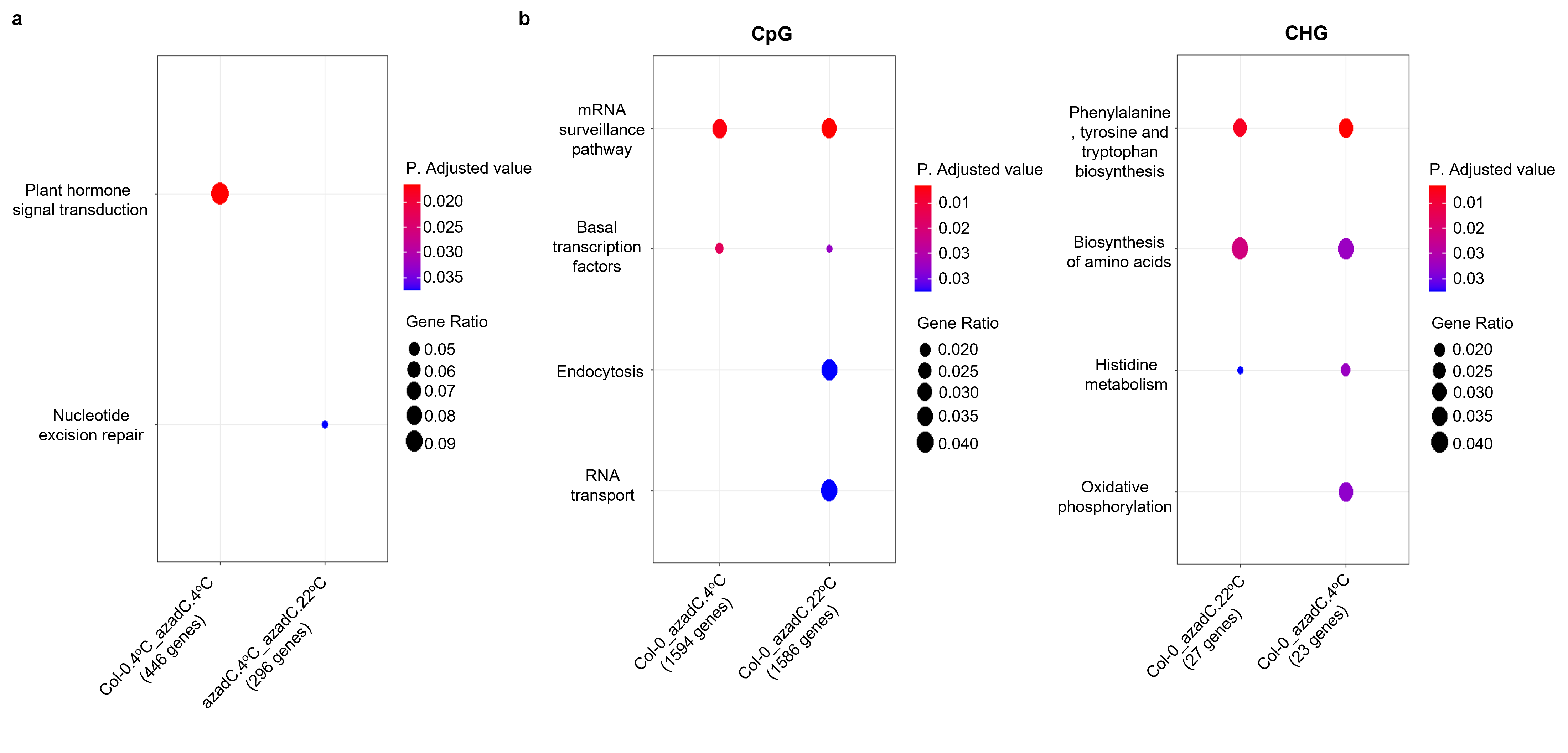

Supplement: Supplementary file 1 [file plants-11-01105-s001.zip › Figure S5.png]
